# Brain natriuretic peptide to predict successful liberation from mechanical ventilation in critically ill patients: a systematic review and meta-analysis

**DOI:** 10.1186/s13054-020-2823-9

**Published:** 2020-05-11

**Authors:** Jean Deschamps, Sarah K. Andersen, Jordan Webber, Robin Featherstone, Meghan Sebastianski, Ben Vandermeer, Janek Senaratne, Sean M. Bagshaw

**Affiliations:** 1 Department of Critical Care Medicine, Faculty of Medicine and Dentistry, University of Alberta, 8440 112 St. NW, Critical Care Medicine 2-124E Clinical Sciences Building, Edmonton, Alberta T6G 2B7 Canada; 2 Alberta Strategy for Patient Oriented Research (SPOR) SUPPORT Unit—Knowledge Translation Platform, University of Alberta, 4-472 Edmonton Clinical Health Academy, 11405-87 Avenue, Edmonton, Alberta T6G 1C9 Canada; 3 Alberta Research Center for Health Evidence (ARCHE), University of Alberta, 4-486D Edmonton Clinical Health Academy, 11405-87 Avenue, Edmonton, Alberta T6G 1C9 Canada; 4 Knowledge Translation Platform, Alberta SPOR SUPPORT Unit Department of Pediatrics, University of Alberta, 362-B Heritage Medical Research Centre (HMRC), Edmonton, Alberta Canada; 5 Division of Cardiology, Faculty of Medicine and Dentistry, University of Alberta, 8440 112 St. NW, Edmonton, Alberta Canada

**Keywords:** Natriuretic peptide (brain), Respiration (artificial), Ventilator weaning, Intensive care, Critical care

## Abstract

**Background:**

Predicting successful liberation from mechanical ventilation (MV) in critically ill patients is challenging. Brain natriuretic peptide (BNP) has been proposed to help guide decision-making for readiness to liberate from MV following a spontaneous breathing trial (SBT).

**Methods:**

We performed a systematic review and meta-analysis of randomized and prospective observational studies that measured BNP levels at the time of SBT in patients receiving MV. The primary endpoint was successful liberation from MV (absence of reintubation or non-invasive ventilation at 48 h). Statistical analyses included bi-variate and Moses-Littenberg models and DerSimonian-Laird pooling of areas under ROC curve (AUROC).

**Results:**

A total of 731 articles were screened. Eighteen adult and 2 pediatric studies were fulfilled pre-specified eligibility. The measure of the relative variation of BNP during SBT (ΔBNP%) after exclusion of SBT failure by clinical criteria in adults yielded a sensitivity and specificity of 0.889 [0.831–0.929] and 0.828 [0.730–0.896] for successful liberation from MV, respectively, with a pooled AUROC of 0.92 [0.88–0.97]. The pooled AUROC for any method of analysis for absolute variation of BNP (ΔBNP), pre-SBT BNP, and post-SBT BNP were 0.89 [0.83–0.95], 0.77 [0.63–0.91], and 0.85 [0.80–0.90], respectively.

**Conclusion:**

The relative change in BNP during a SBT has potential value as an incremental tool after successful SBT to predict successful liberation from MV in adults. There is insufficient data to support the use of BNP in children or as an alternate test to clinical indices of SBT, or the use of ΔBNP, BNP-pre, and BNP-post as an alternate or incremental test.

**Trial registration:**

PROSPERO CRD42018087474 (6 February 2018)

## Take home message

The relative change in BNP during a SBT (ΔBNP%) may be a useful and incremental tool for patients who pass their SBT to predict the successful liberation from mechanical ventilation; however, further research is needed. There is presently insufficient data to support the use of BNP as a stand-alone test compared to clinical indices of SBT or to support the use of additional measures of BNP (i.e., ΔBNP, BNP-pre SBT, and BNP-post SBT) as either a stand-alone or incremental tool.

### Tweet

The relative change in BNP may be a useful tool after clinical SBT success to predict liberation from mechanical ventilation.

## Introduction

Predicting successful liberation from mechanical ventilation (MV) among critically ill patients can be challenging, and there are no standardized methods for assessing readiness for extubation [1]. The American College of Chest Physicians/American Thoracic Society (ACCP/ATS) clinical practice guideline (CPG) on liberation from MV suggests a spontaneous breathing trial (SBT) with inspiratory pressure support as the preferred technique; however, this recommendation is based on limited evidence [1, 2]. Moreover, the majority of parameters used to determine whether SBT has been successful are physiologic variables that inconsistently predict successful liberation from MV [3–5]. Brain natriuretic peptide (BNP) has been proposed as a novel biomarker to help predict successful liberation from MV. To date, CPGs have not integrated evidence from studies evaluating BNP to predict successful liberation from MV.

BNP is a sensitive marker of myocardial stretch, and its relative change in patients during a SBT has been proposed to provide incremental value to predict successful liberation from MV [3, 5]. BNP is a natriuretic peptide released from cardiomyocytes, measured using one of the two widely available assays (NT-proBNP and BNP). The half-life of BNP is estimated to be 20 min, while the half-life of NT-proBNP is estimated at 120 min [3]. Subclinical congestion and overt pulmonary edema due to changes in left ventricular afterload may be common during a SBT. These physiologic changes may be readily detected by measuring changes in BNP [5–9]. Existing studies have attempted to incorporate BNP at various steps of liberation from MV. The focus of this study will be the use of BNP during a SBT.

Accurate and reliable prediction of extubation failure is clinically important, as extubation failure is known to have greater risk of adverse outcomes including reintubation, nosocomial pneumonia, mortality, and prolonged length of intensive care unit (ICU) stay [2, 10–13]. Development and validation of rigorous methods incorporating BNP (both BNP and NT-proBNP will be hereto referred to as BNP for the purpose of this manuscript) may augment clinician decision support and rates of successful liberation from MV and improve patient outcomes. The objective of this systematic review was to rigorously evaluate the value of BNP measurement with a SBT as a biomarker to predict liberation from MV among critically ill ICU patients. We hypothesized that BNP would add incremental predictive value for successful liberation from MV compared with standard clinical and biochemical parameters assessed during SBT.

## Methods

The systematic review protocol has been registered with the PROSPERO International Prospective Register of Systematic Reviews (registration number CRD42018087474 on 6 February 2018) and has been published (12 February 2019). Data were sourced from available published and unpublished studies. As such, no patient-specific primary data were collected and research ethics approval was not required.

### Search strategy and study identification

#### Search methods

The search strategy was developed and executed by a research librarian (RF) and was peer-reviewed by a second research librarian (Additional file 1) [14–18]. We searched electronic databases: Ovid MEDLINE (1946 to present); Ovid EMBASE (1974–present); Wiley Cochrane Library (inception–present), including the Cochrane Database of Systematic Reviews (CDSR) and the Cochrane Central Register of Controlled Trials (CENTRAL); and Web of Science Core Collection via Clarivate Analytics (1900–present). A combination of the following search themes was used: (1) brain natriuretic peptide, any subtype, and (2) weaning, extubation, or liberation from mechanical ventilation. Results were limited to human studies, published in any language from database inception. Bibliographic records were exported to an EndNote X7 (Thomson Reuters, Philadelphia, PA) database for duplicate removal and screening. Additional sources were included in the search strategy. The cited and citing references of included studies and relevant review articles were screened. We also searched trial registry records via ClinicalTrials.gov and meeting abstracts via the Conference Proceedings Citation Index (Clarivate Analytics). Finally, we identified relevant clinical practice guidelines by searching Choosing Wisely Canada, the National Guidelines Clearinghouse, and TRIP (Turning Research Into Practice) Database.

#### Study assessment

We included all relevant randomized and pseudo-randomized controlled trials (defined as controlled trials in which patients are randomized according to methods other than concealed random allocation) that measured BNP levels at the time of SBT in patients receiving MV. We also included prospective observational studies that described BNP levels during SBT and assessed for any association with successful extubation rates. We excluded retrospective studies since the timing of BNP measurement relative to SBT was of critical importance for the purpose of this study and may have been prone to bias. We included studies reported as full text, published as an abstract only, and any relevant unpublished data obtained from the authors. There was no language restriction.

Studies were included if they involved patients receiving invasive MV in whom SBT was performed. There were no age restrictions. We included studies with BNP assay of any type (BNP, NT-proBNP, etc.) if performed within 120 min of the SBT. There were no restrictions on SBT type. We excluded studies with insufficient data for the outcomes measured when we were unable to obtain the necessary original data from the primary authors.

Eligible articles were identified through two phases. In the first phase, two authors (JD and JW) independently reviewed the titles and abstracts of all retrieved bibliographic records using EndNote X7 (Thomson Reuters, Philadelphia, Pennsylvania) for potential inclusion. In the second phase, full texts of the selected articles were retrieved and two authors (JD and JW) independently reviewed and selected studies that met the inclusion criteria.

#### Data extraction

For full-text studies selected for inclusion, relevant information was abstracted using piloted and standardized electronic data forms by two authors independently (JD and SA) (Additional file 2). Abstracted data was then compared between the two authors. Disagreements at every step were resolved through discussion and a third author (SMB) was available for arbitration.

### Data analysis and synthesis

The primary endpoint was successful liberation from MV. Successful liberation was defined in accordance with existing literature as the absence of reintubation or application of new non-invasive ventilation in the 48 h following initial extubation [1]. We further analyzed additional data after 48 h as available. Secondary endpoints initially described in our protocol could not be analyzed due to insufficient available data.

We examined and compared the characteristics of patients that had failed extubation compared to those that had successful extubation. For binary variables (e.g., sex) we computed odds ratio, while for continuous variables (e.g., age), we computed mean differences. When results from multiple studies were available, we pooled data using a DerSimonian-Laird random effects model. All results were presented with 95% confidence intervals. Heterogeneity was examined using the *I*-squared statistic.

Diagnostic accuracy of BNP was measured using sensitivity and specificity. In this instance, a true positive was represented by the combination of a low absolute or low variation of BNP in a patient that had successful liberation from MV. A true negative was represented by the combination of a high absolute or high variation of BNP in a patient that had unsuccessful liberation from MV. Where possible, results across studies were simultaneously pooled using a bi-variate model to create both a joint estimate of sensitivity and specificity (with 95% confidence regions) as well as hierarchical summary ROC curve (HSROC). For subgroup analyses, we were unable to use the bi-variate model due to insufficient studies. In these cases, the Moses-Littenberg model was used to estimate a summary ROC curve. When considered sufficiently clinically homogeneous, areas under the ROC curve (AUROC) were pooled when possible using a DerSimonian-Laird random effects model.

### Quality assessment

Quality of each study (JD and SA) was assessed using the QUADAS-2 questionnaire for systematic reviews [19]. Ten parameters across four domains (patient selection, index test, reference test, and flow/timing) were analyzed independently by two authors (JD and SA) and disagreements resolved through discussion. This was performed in duplicate by two independent reviewers (JD and SA). We applied the strict QUADAS-2 method of assigning low risk or at risk status for each domain. We did not perform a GRADE assessment as described in the protocol, as this was deemed not applicable to this type of research.

## Results

**Fig. 1:**
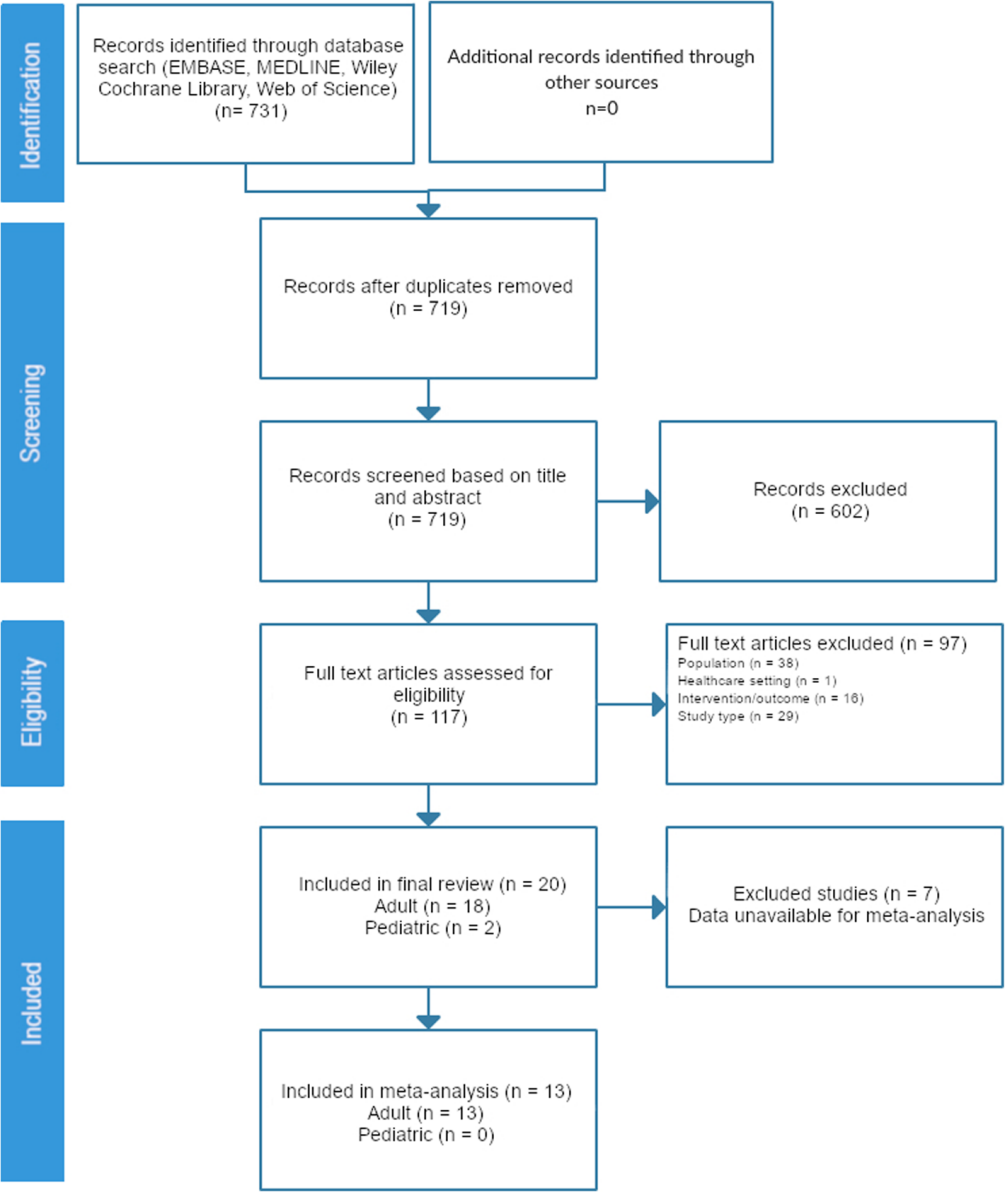
Flow diagram for study assessment. This diagram illustrates the flow of study selection for the systematic review and meta-analysis

A total of 731 articles were screened, 117 were identified for full-text review, and 20 met the pre-specified eligibility criteria and were included (Fig. 1, Additional file 3). Of these, 18 studies were of adult patients and 2 studies were of children (Tables 1 and 2).

**Table 1:** Included adult studies

Author	Total patients	Population	Heart disease	Renal failure	SBT type	SBT failure status	BNP type	BNP measure	Dichotomization threshold
Cheng L (2015) (Additional file 3)	56	Mixed population	Excluded	Excluded	PS/PEEP	Exclude SBT failure	BNP	ΔBNP, ΔBNP%	80 ng/L, 13.4%
Chien et al. [4]	52	Mixed population	Included	Included	T-tube	Exclude SBT failure	BNP	ΔBNP%	20%
Fang M (2010) (Additional file 3)	126	Septic shock	Included	Unclear	PEEP	Include SBT failure	NT-proBNP log NT-proBNP	Pre-SBT BNP	3914.5 ng/L
Fang M (2013) (Additional file 3)	52	Septic shock	Included	Unclear	PEEP	Include SBT failure	NT-proBNP log NT-proBNP	Pre-SBT BNP	N/A
Farghaly S (2015) (Additional file 3)	30	Chronic respiratory failure	Excluded	Excluded	PS/PEEP	Include SBT failure	BNP	Post-SBT BNP ΔBNP%	164 ng/L, 14.9%
Haji K (2018) (Additional file 3)	53	Mixed population	Included	Included	PEEP	Include SBT failure	BNP	BNP-post	N/A
Hersh D (2004) (Additional file 3)	23	Mixed population	Unclear	Unclear	Unclear	Exclude SBT failure	BNP	Pre-SBT BNP	N/A
Konomi I (2016) (Additional file 3)	42	Mixed population	Included (except valvulopathy)	Included	T-piece	Include SBT failure	BNP	Pre-SBT BNP	N/A
Lara TM (2013) (Additional file 3)	101	Elective CABG with CPB	Included	Excluded (CKD)	Unclear	Include SBT failure	BNP	Post-SBT BNP	299 ng/L
Luo L (2017) (Additional file 3)	60	Mixed population (failed extubation once)	Included (except MV disease)	Included	T-tube	Exclude SBT failure	NT-proBNP	Post-SBT BNP	N/A
Ma G (2013) (Additional file 3)	29	Cancer patients with pulmonary complications undergoing noncardiac major surgery	Excluded	Excluded	T-tube	Include SBT failure	NT-proBNP	Post-SBT BNP	448 ng/L
Maraghi SE (2014) (Additional file 3)	40	Mixed population	Excluded	Excluded	T-tube	Exclude SBT failure	BNP	ΔBNP%	20%
Martini A (2011) (Additional file 3)	98	Unclear	Included	Unclear	Unclear	Unclear	NT-proBNP	ΔBNP	N/A
Mekontso Dessap et al. [5]	102	Mixed population	Included	Excluded	T-Piece PS/PEEP	Include SBT failure	BNP	Pre-SBT BNP, ΔBNP	N/A
Ouanes-Besbes L (2012) (Additional file 3)	143	Mixed population	Included	Excluded (any)	T-tube	Exclude SBT failure	NT-proBNP	Pre-SBT BNP	Rule-in, 2000 ng/L; rule-out, 1000 ng/L
Soummer A (2012) (Additional file 3)	100	Mixed population	Included	Included	T-tube	Exclude SBT failure	BNP	Post-SBT BNP	267 ng/L
Wang YT (2016) (Additional file 3)	82	Mixed population	Excluded	Excluded	PEEP	Exclude SBT failure	NT-proBNP	Post-SBT BNP	N/A
Zapata et al. [11]	100	Mixed population	Included	Included	T-tube	Exclude SBT failure	BNP NT-proBNP	Pre-SBT BNP, ΔBNP	BNP, 263 ng/L; NT-ProBNP, 1343 ng/L; ΔBNP, 48 ng/L; ΔNT-ProBNP, − 21 ng/L

**Table 2:** Included pediatric studies

Author	Total patients	Population	Heart disease	Renal failure	SBT type	SBT failure status	BNP type	BNP measure	Dichotomization threshold
Flint J (2012) (Additional file 3)	20	Congenital cardiac surgery	Included	Unclear	PS/PEEP	Exclude SBT failure	BNP	BNP-pre, BNP-post	N/A
Zhang Q (2014) (Additional file 3)	88	Respiratory distress syndrome	Excluded	Excluded	T-tube	Include SBT failure	BNP	BNP-pre	18,500 ng/L

### Adult studies

**Fig. 2:**
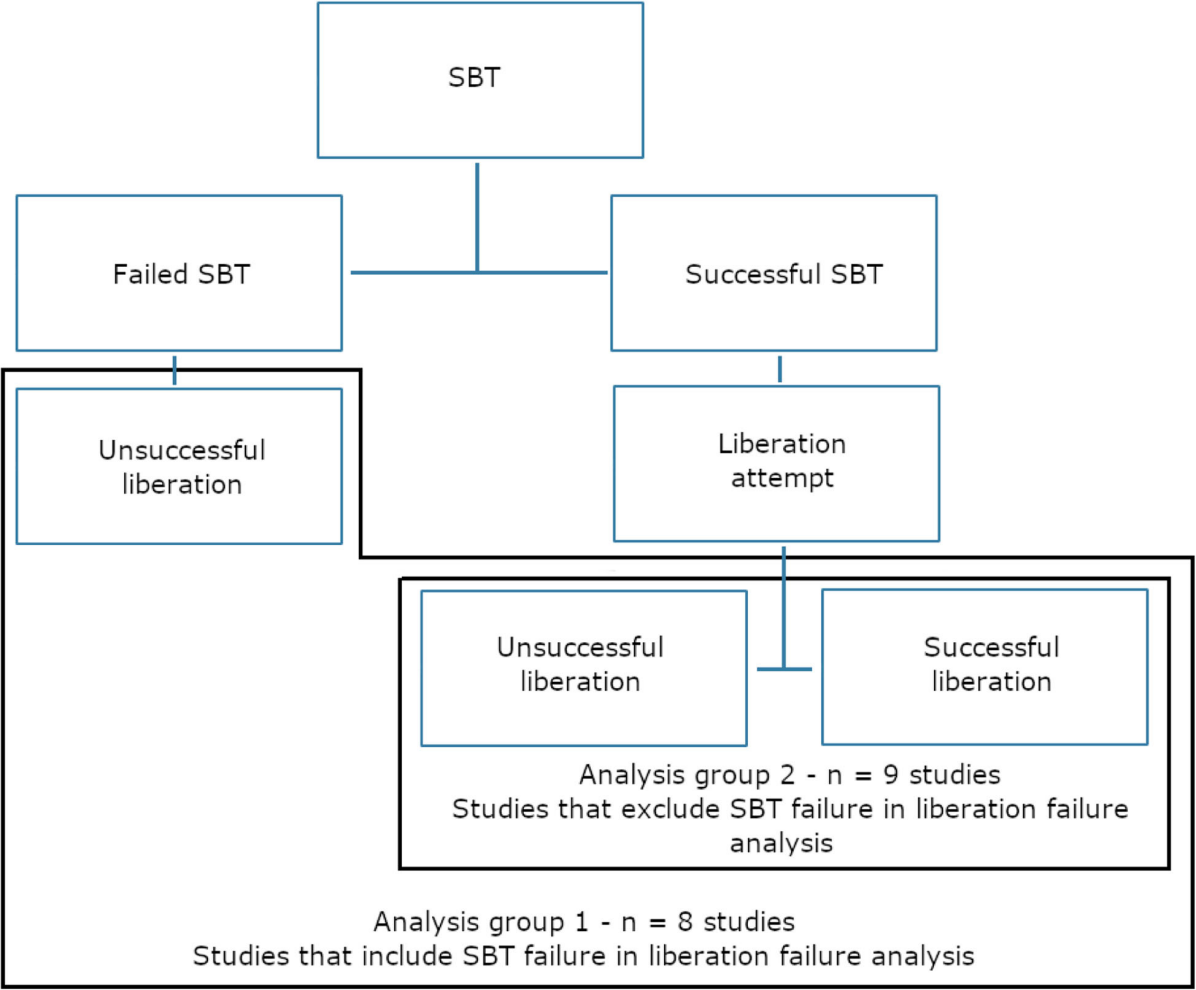
Flow diagram of group allocation for inclusion versus exclusion of SBT failure in the liberation failure analysis. SBT: Spontaneous breathing trial; successful liberation: absence of non-invasive ventilation or reintubation at 48h; unsuccessful liberation: failure of SBT or non-invasive ventilation or reintubation at 48h. This flow diagram details how the different stages at which unsuccessful liberation from mechanical ventilation were handled in regard to statistical analysis. Group 1 included SBT failure within the unsuccessful liberation umbrella for analysis of liberation failure. Group 2 excluded SBT failure from the umbrella of unsuccessful liberation for the purpose of analysos of liberatio failure

Of the 18 adult studies, there were 28 individual analyses of BNP relative to SBT (Table 1). The methods of BNP measurements included pre-SBT BNP measures (BNP-pre), post-SBT BNP measures (BNP-post), absolute BNP change during SBT (ΔBNP), and the relative BNP change during SBT (ΔBNP% = [post-SBT BNP − pre-SBT BNP]/pre-SBT BNP). Four studies described more than one measure of BNP. Some studies did not provide a ROC analysis and relied on odds ratio (OR). A subset of studies assessed BNP as an alternative tool to reclassify all patients by including SBT failure in the liberation failure group for analysis (group 1; Fig. 2). The other subset assessed BNP as an incremental tool in addition to SBT testing and excluded SBT failure from the analysis (group 2; Fig. 2). This was unclear in one study [20]. All studies relied on clinical indices of SBT to determine readiness of extubation, and no decision to extubate was done based on BNP measures. All studies excluded preventative non-invasive ventilation from the cohort of patients being analyzed.

Patient baseline characteristics, primary diagnoses, acuity scores, and ventilatory parameters were reported by status of liberation from MV (Additional file 4). These baseline data points were not classified according to BNP assessment results combined. All studies except one [19] evaluated the first liberation attempt (*n* = 17). All studies observed patients for extubation failure at 48 h (*n* = 18), with one study extending observation to 7 days [19]. The quality of included studies focused on adults suggested a risk of bias for all but one low-risk study [21] (Table 3). The main reasons for risk of bias were patient selection and lack of blinding of the index test and reference test.

**Table 3:** Quality assessment of adult studies

Authors	Risk of Bias		Reference test	Flow/timing	Applicability	Index test	Reference test	Total quality	Applicability
	Patient selection	Index test			Patient selection				
Cheng L (2015) (Additional file 3)	At risk	At risk	At risk	Low	No	No	No	At risk	No concerns
Chien et al. [4]	At risk	At risk	At risk	Low	No	No	No	At risk	No concerns
Chien et al. [4]	At risk	Low	At risk	Low	No	No	No	At risk	No concerns
Fang M (2013) (Additional file 3)	At risk	At risk	At risk	Low	No	No	No	At risk	No concerns
Fang M (2010) (Additional file 3)	At risk	At risk	At risk	Low	No	No	No	At risk	No concerns
Farghaly S (2015) (Additional file 3)	At risk	At risk	At risk	Low	No	No	No	At risk	No concerns
Haji K (2018) (Additional file 3)	At risk	At risk	At risk	Low	No	No	No	At risk	No concerns
Hersh D (2004) (Additional file 3)	At risk	At risk	At risk	At risk	No	No	No	At risk	No concerns
Konomi I (2016) (Additional file 3)	Low	At risk	Low	Low	No	No	No	At risk	No concerns
Lara TM (2013) (Additional file 3)	Low	At risk	At risk	Low	No	No	No	At risk	No concerns
Luo L (2017) (Additional file 3)	At risk	At risk	At risk	Low	No	No	No	At risk	No concerns
Ma G (2013) (Additional file 3)	Low	Low	Low	Low	No	No	No	At risk	No concerns
Maraghi SE (2014) (Additional file 3)	Low	At risk	At risk	Low	No	No	No	At risk	No concerns
Martini A (2011) (Additional file 3)	At risk	At risk	At risk	At risk	No	No	No	At risk	No concerns
Mekontso Dessap et al. [5]	Low	At risk	Low	Low	No	No	No	At risk	No concerns
Ouanes-Besbes L (2012) (Additional file 3)	Low	At risk	At risk	Low	No	No	No	At risk	No concerns
Soummer A (2012) (Additional file 3)	Low	Low	Low	Low	No	No	No	Low	No concerns
Wang YT (2016) (Additional file 3)	Low	At risk	At risk	At risk	No	No	No	At risk	No concerns
Zapata et al. [11]	At risk	At risk	Low	Low	No	No	No	At risk	No concerns
Flint J (2012) (Additional file 3)	At risk	At risk	Low	At risk	No	No	No	At risk	No concerns
Zhang Q (2014) (Additional file 3)	At risk	At risk	Low	At risk	No	No	No	At risk	No concerns

### Pediatrics studies

Of the two studies focused on children, one study addressed preterm infants with respiratory distress syndrome, and the other addressed congenital heart surgery patients (Table 2). Pooled analysis could not be performed due to the lack of available data. As such, these studies were excluded from the pooled analysis (Table 3). Both studies were at risk of bias (Table 4).

**Table 4:** Quality assessment of pediatric studies

Authors	Risk of bias		Reference test	Flow/timing	Applicability	Index test	Reference test	Total quality	Applicability
	Patient selection	Index test			Patient selection				
Flint J (2012) (Additional file 3)	At risk	Low	Low	Low	Low	Low	Low	At risk	No concerns
Zhang Q (2014) (Additional file 3)	At risk	At risk	At risk	Low	Low	Low	Low	At risk	No concerns

### Meta-analysis

**Fig. 3:**
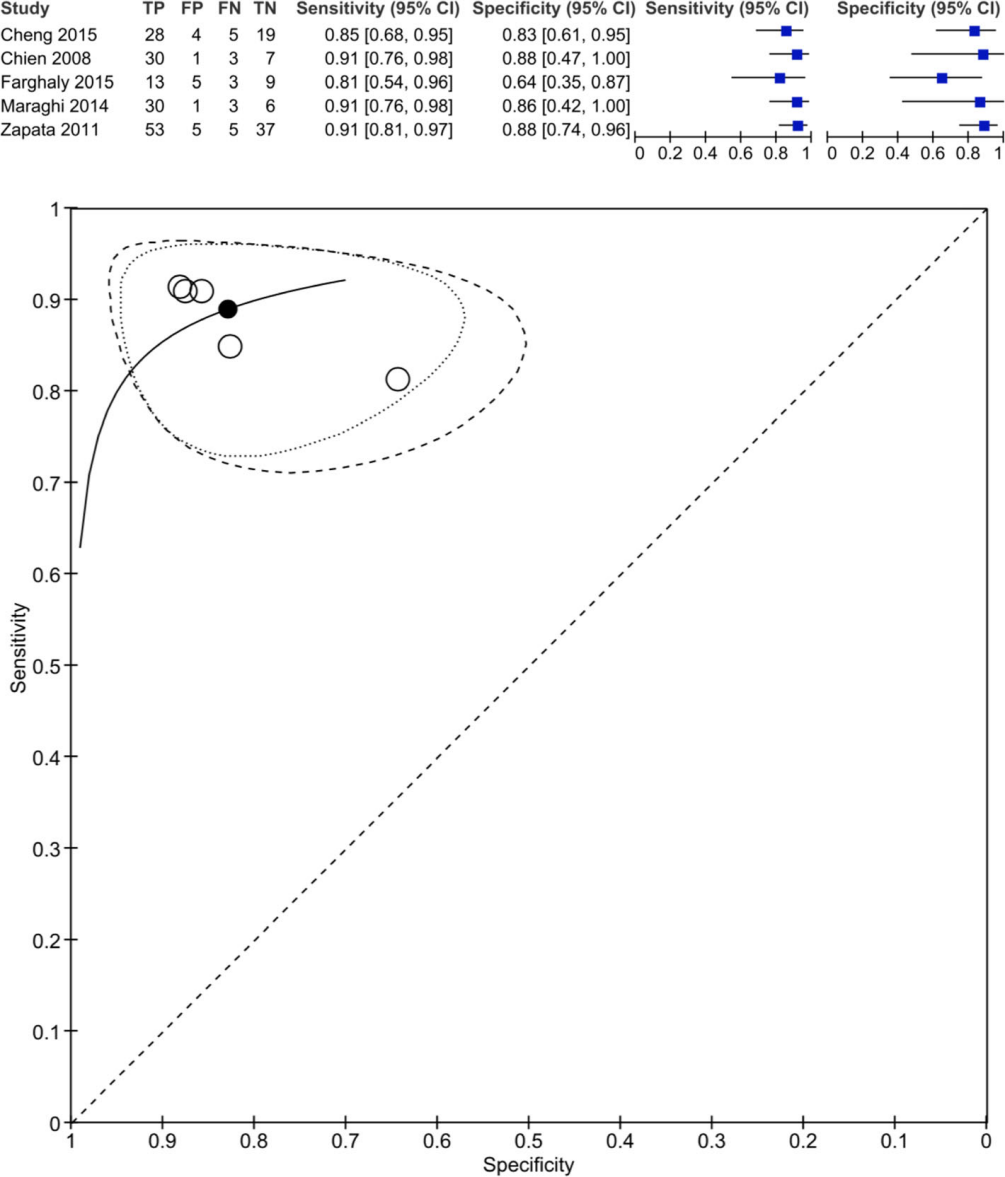
Bi-variate estimate of sensitivity and specificity for measures of either ∆BNP or ∆BNP% in studies that included SBT failure (group 1) and studies that excluded SBT failure (group 2) from liberation failure analysis. TP: True positive; TN: True negative; FP: False positive; FN: False negative. This diagram illustrates the bi-variate analysis performed on 5 studies and provides an estimate of sensitivity and specificity for studies regardless of the method of BNP measurement (DBNP or DBNP%) and regardless of inclusion or exclusion of SBT failure from the liberation failure analysis (group 1 or 2). This was obtained through analysis of the TP, TN, FP and FN obtained in studies that provided full description of their population and outcomes

Only 13 of the 20 studies included had sufficient data for pooled meta-analysis. We performed a single bi-variate estimate of sensitivity and specifity combining studies that reported absolute and relative changes of BNP (ΔBNP or ΔBNP%) in studies that either included or specifically excluded patients with SBT failure from analysis (*n* = 5). The sensitivity and specificity were 0.889 (0.831–0.929) and 0.828 (0.730–0.896), respectively (Fig. 3). This was further stratified in Moses-Littenberg summary ROC curves for either measure of BNP (ΔBNP or ΔBNP%) and for ΔBNP% only in studies that excluded patients with SBT failure (Additional file 5).

**Fig. 4:**
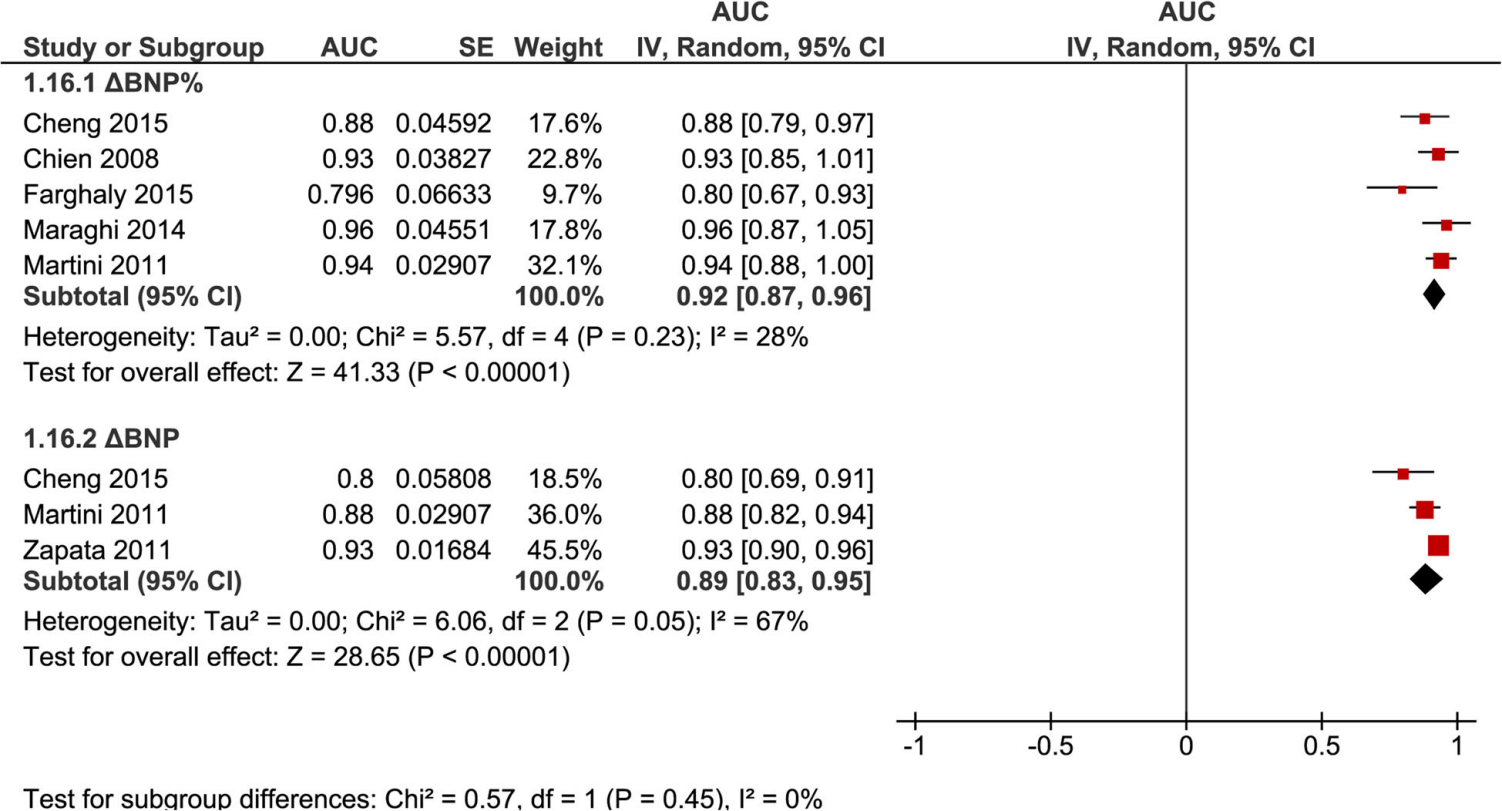
Pooled diagnostic AUC for either ∆BNP or ∆BNP% methods of measurements, in studies that included SBT failure (group 1) and studies that excluded SBT failure (group 2) from liberation failure analysis. These figures illustrate the individual AUC obtained from the studies for each method of BNP measurement separately (DBNP and DBNP%). Both studies that included SBT failure (group 1) and excluded SBT failure (group 2) in liberation failure analysis were included to increase statistical power. The pooled AUC for DBNP% showed a high AUC with low heterogeneity, while the pooled AUC for DBNP showed a high AUC with moderate heterogeneity

**Fig. 5:**
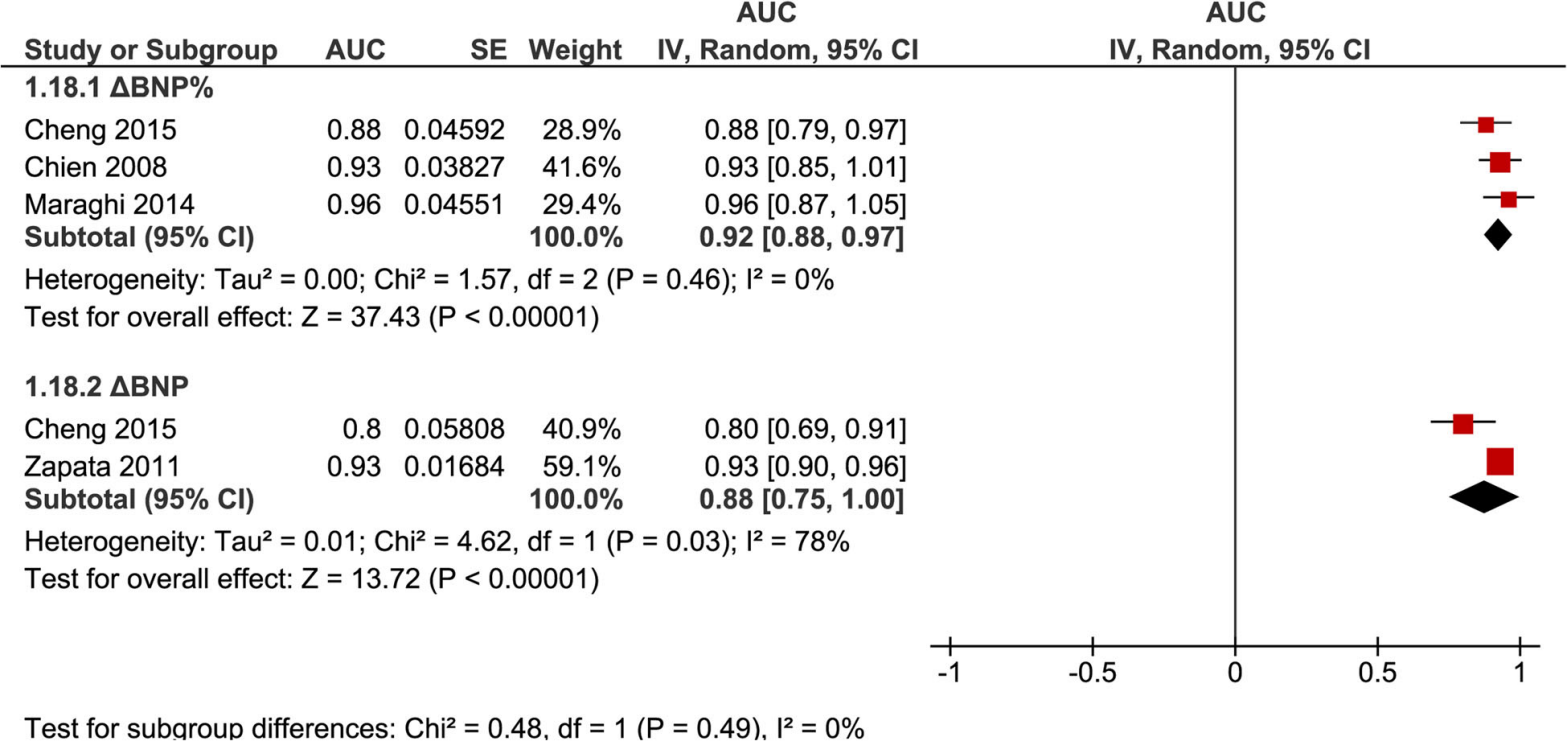
Pooled diagnostic AUC either ∆BNP or ∆BNP% methods of measurements in studies that excluded SBT failure (group 2) from liberation failure analysis. These figures illustrate the individual AUC obtained from the studies for each method of DBNP and DBNP% measurements separately. Only studies that excluded SBT failure (group 2) in liberation failure analysis were included to provide more precise data of a specific clinical subgroup. The pooled AUC for DBNP% showed a high AUC with low heterogeneity. The pooled AUC for DBNP showed a high AUC and high heterogeneity but is limited by the inclusion of only 2 studies in the analysis

**Fig. 6:**
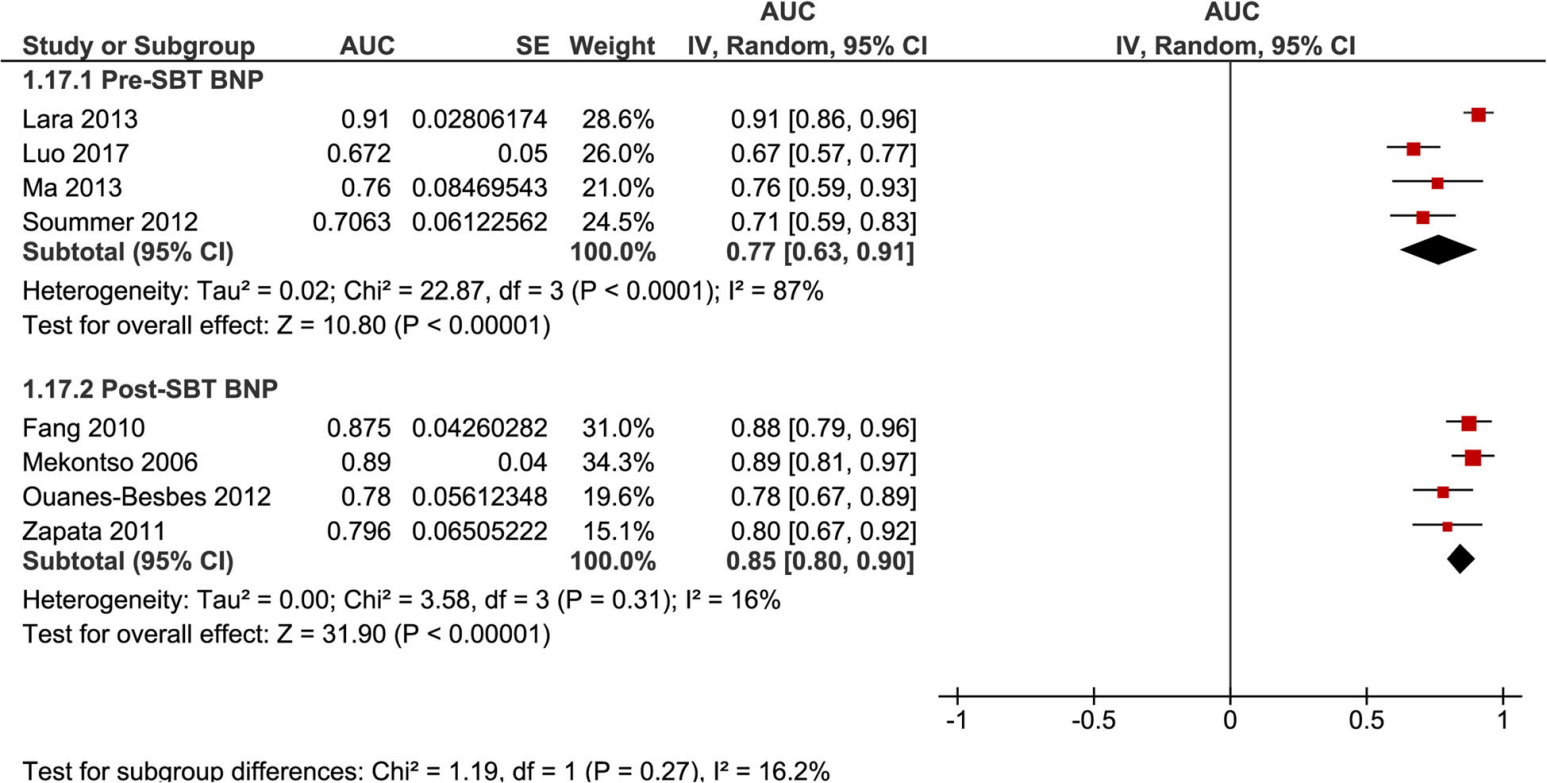
Pooled diagnostic AUC of either BNP-pre or BNP-post methods of measurement in studies that included SBT failure (group 1) and studies that excluded SBT failure (group 2) from liberation failure analysis. These figures illustrate the individual AUC obtained from the studies for each method of individual measures of BNP separately (BNP-pre and BNP-post). Both studies that included SBT failure (group 1) and excluded SBT failure (group 2) in liberation failure analysis were included to increase statistical power. The pooled AUC for BNP-pre showed a moderatehigh AUC with high heterogeneity. The pooled AUC for BNP-post showed a high AUC and low heterogeneity

AUROC were pooled separately for ΔBNP (*n* = 3, 0.89 [0.83–0.95], *I*^2^ = 67%) and ΔBNP% (*n* = 5, 0.92 [0.87–0.96], *I*^2^ = 28%) regardless of inclusion or exclusion of patients failing a SBT (Fig. 4); for ΔBNP% for studies that excluded patients failing a SBT (*n* = 3, 0.92 [0.88–0.97], *I*^2^ = 0%) (Fig. 5); and for BNP-pre (*n* = 4, 0.77 [0.63–0.91], *I*^2^ = 87%) and BNP-post (*n* = 4, 0.85 [0.80–0.90], *I*^2^ = 16%) regardless of inclusion or exclusion of patients failing a SBT (Fig. 6).

No meta-analysis of the specific thresholds of BNP measures could be performed due to insufficient data. A net reclassification index could not be calculated given the limited data assessing BNP measures in SBT failure groups. Only the pediatric study by Zhang et al. [22] calculated a NRI of 0.224 for the addition of NT-ProBNP to SBT, suggesting an improvement in reclassification.

## Discussion

### Key findings

This meta-analysis supports the validity of the relative change of BNP (ΔBNP%) during a SBT to add incremental value and inform the likelihood of successful liberation from MV in adults. This meta-analysis also demonstrated high accuracy using a pooled AUROC of ΔBNP% for studies that excluded patients who failed a SBT. Combining methods of absolute and relative change in BNP measures, irrespective of inclusion or exclusion of patients who failed their SBT showed high sensitivity and specificity for predicting successful liberation. The data from pediatric studies and studies describing other BNP measures, such as ΔBNP, BNP-pre SBT, and BNP-post SBT, were insufficient to suggest incremental value and for clinical decision support about likelihood of liberation success.

There findings are noteworthy given the limited predictive ability of SBT alone, which is generally regarded as the best available assessment. In studies, SBT misclassified patients in 10–20% of patients who subsequently failed successful liberation from MV [1, 12]. While reintubation was described as occurring without immediate difficulty, these patients had greater risk of morbidity and mortality following a failed attempt at liberation from MV [1]. Better prediction by use of alternative tests that add incremental value, such as BNP, may lead to greater confidence in clinical decision-making to extubate, reduced reintubation, and improved outcomes. This possibility has been recognized as early as 2008 in two methods of analysis: as an incremental “value-added” test during SBT [3] and as a “stand-alone” alternative test [5]. These two approaches were well represented in the studies included in this meta-analysis. A subset of studies (group 1, *n* = 8) included patients failing their SBT in the analysis of the group that failed MV liberation; this in effect assessed BNP as an alternative test to conventional SBT. This method may have decreased accuracy compared to evaluation of pooled analysis where patients who failed their SBT were excluded. A second subset of studies (group 2, *n* = 9) excluded patients with a failed SBT in the analysis of the MV liberation failure group. This in effect assesses BNP as an incremental test to a successful SBT. The major benefit in this case is the potential reclassification of patients for whom liberation may have been attempted but may have failed. This distinction is important to determine the optimal use of BNP in assessment for MV liberation. In our view, the two ways in which SBT failure is incorporated in the analysis should ideally be pooled and analyzed separately. However, we expected limited data and pooled them for further analysis as planned in the protocol. Similarly, the different methods of BNP measures (ΔBNP, DeltaBNP%, BNP-pre, and BNP-post) should not be pooled, as some address a change in BNP values, whereas others only address a single value at a specific time. The only exception in which BNP measures could be pooled would be ΔBNP and ΔBNP% given the possibility that baseline BNP level may not be relevant in the case of substantial change occurring during a SBT.

Most of the data that could be pooled related to absolute and relative changes in BNP during a SBT (ΔBNP and ΔBNP%). In order to increase the breadth of our analyses, we pooled studies of either method of measures (ΔBNP and ΔBNP%) for studies that excluded patients who failed their SBT. The Moses-Littenberg summary ROC analysis showed high accuracy (Additional file 5). However, this analysis requires the availability of true-positive, true-negative, false-positive, and false-negative data to perform, limiting its applicability to certain subgroups. This summary ROC analysis was mostly driven by ΔBNP% (3 out of 4 studies, 148 out of 178 patients). We were able to perform a pooled AUROC of ΔBNP% for studies that excluded SBT failure from the analysis of the liberation failure (Fig. 5). This AUROC analysis further supports the initial findings and provides evidence of high accuracy (0.92 [0.88–0.97], *I*^2^ 0%). This represents the most robust combination of BNP measures in pooled analysis obtained from the data.

Unfortunately, the data was insufficient to perform sensitivity and specificity estimates for this specific combination of BNP measures. The closest approximation was obtained by a bi-variate analysis using pooled data of studies of either ΔBNP or ΔBNP% measures, regardless of inclusion or exclusion of the SBT failure group. The sensitivity and specificity obtained were high [0.889 (0.831–0.929) and 0.828 (0.730–0.896), respectively]. It is important to note that these results were mostly driven by studies, where patients with SBT failure were excluded (4 out of 5 studies; 248 out of 278 patient s), and ΔBNP% (4 out of 5 studies; 178 out of 278 patients). This closely approximates the prior analysis performed on ΔBNP% for studies that excluded patients with SBT failure from the analysis.

There were insufficient studies to analyze ΔBNP, BNP-pre, and BNP-post separately as an incremental test (i.e., excluding SBT failure from the liberation failure analysis) or an alternate “stand-alone” test (i.e., including SBT failure from the liberation failure analysis). Pooling studies of both methods of analysis for each BNP measure appears to support a high accuracy in these cases (Fig. 4). The main limitation is the inability to determine if it is of better use as an incremental or alternate test. Additionally, for BNP-pre and BNP-post, the studies were not described with as much detail as those for studies on ΔBNP and ΔBNP%. Furthermore, it is difficult to determine a superior measure or method, as only two studies directly compared various BNP measures. Both Cheng et al. [23] and Martini et al. [24] compared ΔBNP and ΔBNP%, and both studies suggested ΔBNP% was superior.

From a clinical standpoint, using these measures requires using a specific threshold for dichotomization between likelihood of liberation success versus failure (Table 1). This was determined through analysis of the AUC curve of best sensitivity, specificity, positive and negative predictive values, and diagnostic accuracy. No pre-specified threshold was studied prospectively across any of the studies. Studies of ΔBNP% suggested a threshold above 13.4–20% (*n* = 4) optimally predicted liberation failure, if both BNP types (BNP and NT-ProBNP) were pooled. The other BNP measures had three or fewer studies for each measure or combination (Table 1). As such, this systematic review cannot recommend a specific threshold for any of the BNP measures to optimally discriminate liberation success and failure that could be adopted in clinical practice.

Our study would suggest that BNP performs best if used as a relative change BNP during a SBT among those studies that only included adult patients who successfully passed an initial SBT by other clinical criteria. There is a potential role to the use of either ΔBNP% or ΔBNP irrespective of whether patients have successfully or unsuccessfully passed an SBT, but the data is less robust and requires further investigation.

### Limitations

As described above, the heterogeneity of BNP measures and varying analytic approaches limited our ability to perform pooled analysis; acknowledging this limits the inferences that can be made. Similarly, due to limitation in the reporting across studies, we were unable to perform stratified analysis by potentially important subgroups, such as case-mix and type of ICU admission. We opted to combine both general ICU populations and specific ICU subgroups to capture enough data to perform the AUROC analyses. The bi-variate analysis and Moses-Littenberg analyses were unaffected, as all studies included were of a mixed ICU population. We believe that this makes our results more generalizable to mixed medical/surgical ICU practice; however, we cannot provide strong inferences on the value of BNP for MV liberation in selected ICU subgroups, as each population was represented by a single study. There are several confounders to the accuracy of BNP testing. Heart disease (and specifically depressed left ventricular ejection fraction) and kidney failure can significantly alter BNP kinetics. Unfortunately, these patient characteristics were inconsistently included or excluded across studies. In the case of kidney failure, the distinction between acute and chronic renal failure was also poor. In balance, renal function was normal in most studies, and at most mildly impaired in the rest. As for heart disease, the definitions were variable. The etiology of respiratory failure has an impact on the accuracy of BNP: delirium, traumatic brain injury, inability to clear secretions, or stridor, among others, limit the accuracy of BNP as they may not lead to a change in BNP measurements. A low number of studies that were included in this review attempted to limit this impact by excluding stridor and TBI from the analysis. Unfortunately, capturing clearance of secretions or delirium as the cause of respiratory failure is understandably difficult and was not done in any study. Another limitation is the lack of studies that directly compared the accuracy of a successful SBT by clinical indices and by BNP measure. In this instance, a patient that has passed a SBT by clinical indices may fail by BNP measure, leading to a delay in extubating a patient that would have succeeded. Unfortunately, the relative accuracies were not directly assessed in any study.

Finally, the quality of studies (as defined by QUADAS-2) uniformally ranked as at risk of bias, except for one [21]. The main issue was lack of transparency regarding blinding of physicians to the BNP test. In our opinion, this is not a critical flaw, as the decision to extubate patients was most often based on clinical SBT criteria in all studies.

### Implications for clinical, policy, and research

Research on mechanical ventilation liberation is complex and would benefit from greater standardization. Successful liberation from mechanical ventilation appears well-defined and this is reflected in the studies collected. Liberation failure, on the other hand, has a variable definition among studies, mostly relating to the inclusion or exclusion of SBT failure. Regardless of its importance for applicability of alternative or incremental testing, the terms used require standardization to facilitate research.

Additional data is needed to strengthen BNP as a liberation tool. We consider that this should take the form of a comparative study of BNP as an alternative or an incremental tool to the clinical indices after an SBT. This study would ideally take the form of an assessment of ΔBNP and ΔBNP% and compare inclusion versus exclusion of SBT in the analyzed subgroups. This would allow determination of whether BNP is superior to SBT on its own or simply incremental.

The potential benefits of improved tools to inform greater likelihood of success or failure of liberation from MV have far-reaching implications. On top of reclassifying individuals after initial assessment with clinical indices, this may allow stratification of the risk of failure. Such a stratification may help better determine targets for optimization (such as further volume de-escalation), better identification of the need for post-extubation therapies (such as high-flow oxygen therapy and BiPAP), and need for prolonged ICU observation. Clinical risk scores on this basis could be developed to aid in management of these patients after extubation.

## Conclusion

The relative change of BNP during a SBT (ΔBNP%) would appear to have value as an incremental tool after passing a SBT for predicting successful liberation from MV. This pooled analysis, however, was limited by not enabling calculation or validation of a specific threshold, despite a number of studies reporting thresholds in the range of 13–20%. As such, we submit further investigation is warranted. There was insufficient data to support ΔBNP% as a stand-alone test to conventional SBT or the use of ΔBNP, BNP-pre, and BNP-post either as incrementally or as a stand-alone, alternate, or incremental test. Studies comparing the best use of ΔBNP% either as an alternative or incremental tool to clinical indices during SBT as well as prospective validation of a specific threshold represent the next step in research. There is paucity of data in pediatric cases that limits any conclusion.

## Supplementary information

**Additional file 1.** Search strategy.

**Additional file 2.** List of data extracted from studies.

**Additional file 3.** Adult and pediatric studies included in systematic review reference list.

**Additional file 4.** Baseline patient characteristics comparison between successful and unsuccessful liberation from mechanical ventilation.

**Additional file 5.** Moses-Littenberg analysis of either ΔBNP or ΔBNP% methods of measurements in studies that included (group 1) or excluded SBT failure (group 2) from liberation failure analysis.

**Additional file 6.** Moses-Littenberg analysis of either ΔBNP or ΔBNP% methods of measurements in studies that excluded SBT failure (group 2) from liberation failure analysis.

## Data Availability

This is an open-access article distributed in accordance with the Creative Commons Attribution Non Commercial (CC BY-NC 4.0) license, which permits other to distribute, remix, adapt, build upon this work non-commercially, and license their derivative works on different terms, provided the original work is properly cited and the use is non-commercial.

## Abbreviations

ΔBNP%: Relative variation of BNP during a SBT
MV: Mechanical ventilation
ΔBNP: Absolute variation of BNP during a SBT
BNP: Brain natriuretic peptide
SBT: Spontaneous breathing trial
AUROC: Area under ROC curve
CPG: Clinical practice guideline
ICU: Intensive care unit
HSROC: Hierarchical summary ROC curve
