# Supplementary material for: Brain natriuretic peptide to predict successful liberation from mechanical ventilation in critically ill patients: a systematic review and meta-analysis
Source: Crit Care. 2020 May 11;24:213. doi: 10.1186/s13054-020-2823-9 (PMC7216735; doi:10.1186/s13054-020-2823-9)
Supplement: Supplementary file 1 — Additional file 1. Search strategy. [file 13054_2020_2823_MOESM1_ESM.docx]

Additional file 1 – Search strategy

Database: Ovid MEDLINE(R) Epub Ahead of Print, In-Process & Other Non-Indexed Citations, Ovid MEDLINE(R) Daily and Ovid MEDLINE(R) 1946 to Present

Strategy:

1 Biomarkers/bl [blood] (98008)

2 Natriuretic Peptide, Brain/ (12358)

3 Nerve Tissue Proteins/ (82138)

4 Peptide Fragments/bl [blood] (9609)

5 ((biomarker* or marker*) adj2 (myocardial adj1 (strain* or stretch*))).tw,kf. (15)

6 ((biomarker* or marker*) and (PVR or vascular resistance*)).tw,kf. (837)

7 ((biomarker* or marker*) and (RV strain* or ventricular strain*)).tw,kf. (73)

8 BNP*.tw,kf. (9419)

9 (NT-proBNP* or NTproBNP*).tw,kf. (5224)

10 N terminal proBNP*.tw,kf. (250)

11 natriuretic peptide*.tw,kf. (26470)

12 nerve tissue protein*.tw,kf. (150)

13 RSBI*.tw,kf. (104)

14 shallow breath* ind*.tw,kf. (159)

15 or/1-14 [Combined MeSH & text words for BNP or RSBI] (209479)

16 Ventilator Weaning/ (3436)

17 (extubat* adj2 (fail* or succe* or unsuccessful*)).tw,kf. (1425)

18 CPAP trial*.tw,kf. (59)

19 (pressure support ventilation adj3 trial*).tw,kf. (12)

20 SBT*.tw,kf. (2669)

21 (spontaneous breathing adj3 trial*).tw,kf. (480)

22 ((T-piece* or T-tube*) adj3 trial*).tw,kf. (102)

23 or/16-22 [Combined MeSH & text words for breathing trials] (7088)

24 Airway Management/ (2113)

25 Respiration, Artificial/ (44059)

26 ((airway* or air way*) adj3 (control* or manage*)).tw,kf. (9048)

27 ((artificial* or mechanical*) adj1 (respir* or ventilat*)).tw,kf. (51214)

28 respirator*.tw,kf. (386020)

29 ventilator*.tw,kf. (47484)

30 or/24-29 [Combined MeSH & text words for artificial respiration] (463328)

31 Airway Extubation/ (911)

32 Tidal Volume/ (9117)

33 extubat*.tw,kf. (11387)

34 liberat*.tw,kf. (22786)

35 postextubat*.tw,kf. (485)

36 tidal volume*.tw,kf. (13279)

37 wean*.tw,kf. (42594)

38 or/31-37 [Combined text words for weaning] (92288)

39 and/30,38 [Combined concept for weaning from artificial respiration] (24379)

40 or/23,39 [Combined concepts for breathing trials or weaning from artificial respiration] (28313)

41 and/15,40 [Combined index test & condition concepts] (340)

42 exp Animals/ not Humans/ (4418500)

43 (animal model* or bovine or canine or capra or cat or cats or cattle or cow or cows or dog or dogs or equine or ewe or ewes or feline or goat or goats or horse or hamster* or horses or macaque or macaques or mare or mares or mice or monkey or monkeys or mouse or murine or nonhuman or non-human or ovine or pig or pigs or porcine or primate or primates or rabbit or rabbits or rat or rats or rattus or rhesus or rodent* or sheep or simian or sow or sows or vertebrate or vertebrates).ti. (2157171)

44 41 not (42 or 43) [Excluded animal studies] (308)

45 remove duplicates from 44 (308)
