# Supplementary material for: Brain natriuretic peptide to predict successful liberation from mechanical ventilation in critically ill patients: a systematic review and meta-analysis
Source: Crit Care. 2020 May 11;24:213. doi: 10.1186/s13054-020-2823-9 (PMC7216735; doi:10.1186/s13054-020-2823-9)
Supplement: Supplementary file 2 — Additional file 2. List of data extracted from studies. [file 13054_2020_2823_MOESM2_ESM.docx]

Additional file 2 – List of data extracted from studies

Author

Year

Title

Total patients

SBT type

Duration of SBT

Time between BNP measures

Method of BNP analysis for SBT failure group

Number of patients in SBT failure group

Number of patients extubated

Number of patients in liberation failure group

Number of patients in liberation success group

Age

Sex

Height

Weight

Fluid balance

Ejection fraction (EF)

Creatinine

Hemoglobin

Albumin

Disease severity score

Duration of mechanical ventilation

Respiratory rate

Tidal Volume

Minute ventilation

pH

PaO2

PaCO2

PaO2/FiO2

Number of patients receiving non-invasive ventilation post-extubation

BNP type

BNP measure (BNP-Pre, BNP-post, DeltaBNP, DeltaBNP%)

Positive likelihood ratio (LR+)
Negative likelihood ratio (LR-)

Sensitivity

Specificity

Positive predictive value (PPV)

Negative predictive value (NPV)

Accuracy

Area under curve (AUROC)

Best threshold determined

Odds ratio

Confidence interval
