# Supplementary material for: Brain natriuretic peptide to predict successful liberation from mechanical ventilation in critically ill patients: a systematic review and meta-analysis
Source: Crit Care. 2020 May 11;24:213. doi: 10.1186/s13054-020-2823-9 (PMC7216735; doi:10.1186/s13054-020-2823-9)
Supplement: Supplementary file 3 — Additional file 3. Adult and pediatric studies included in systematic review reference list. [file 13054_2020_2823_MOESM3_ESM.docx]

Additional file 3 – Adult and pediatric studies included in systematic review reference list

1. Cheng L, Jiang L, Wang M, et al (2015) [The value of changes in plasma B-type natriuretic peptide before and after spontaneous breathing trial in predicting weaning outcome in mechanically ventilated patients]. Zhonghua nei ke za zhi 54:486–90
2. Chien J-Y, Lin M-S, Huang Y-CT, et al (2008) Changes in B-type natriuretic peptide improve weaning outcome predicted by spontaneous breathing trial: Critical Care Medicine 36:1421–1426. <https://doi.org/10.1097/CCM.0b013e31816f49ac>
3. Fang M, Chen M, Zheng C, et al (2013) [Clinical value of extravascular lung water and preload parameters in weaning of mechanical ventilation in patients with septic shock]. Zhonghua Wei Zhong Bing Ji Jiu Yi Xue 25:28–31. <https://doi.org/10.3760/cma.j.issn.2095-4352.2013.01.008>
4. Fang M, Hu B, Li H, et al (2010) [Prognostic implication of plasma N-terminal-pro-brain natriuretic peptide in weaning from mechanical ventilation]. Zhongguo Wei Zhong Bing Ji Jiu Yi Xue 22:482–485
5. Farghaly S, Galal M, Hasan AA, Nafady A (2015) Brain natriuretic peptide as a predictor of weaning from mechanical ventilation in patients with respiratory illness. Australian Critical Care 28:116–121
6. Flint J (2013) B-Type natriuretic peptide (BPN): A potential biomarker fore xtubaiton failure in infants following cardiac surgery. Pediatric Critical Care Medicine 14:S96–S118. <https://doi.org/10.1097/PCC.0b013e318292b29c>
7. Haji K, Haji D, Canty DJ, et al (2018) The impact of heart, lung and diaphragmatic ultrasound on prediction of failed extubation from mechanical ventilation in critically ill patients: a prospective observational pilot study. Crit Ultrasound J 10:. <https://doi.org/10.1186/s13089-018-0096-1>
8. Hersh D, Mandell KB, Remolina AM, et al (2004) BNP is Not a Predictor of Successful Extubation. Chest 126:899S. <https://doi.org/10.1378/chest.126.4_MeetingAbstracts.899S>
9. Konomi I, Tasoulis A, Kaltsi I, et al (2016) Left Ventricular Diastolic Dysfunction—An Independent Risk Factor for Weaning Failure from Mechanical Ventilation. Anaesthesia and Intensive Care 44:466–473. <https://doi.org/10.1177/0310057X1604400408>
10. Lara TM, Hajjar LA, de Almeida JP, et al (2013) High levels of B-type natriuretic peptide predict weaning failure from mechanical ventilation in adult patients after cardiac surgery. Clinics (Sao Paulo, Brazil) 68:33–8
11. Luo L, Li Y, Chen X, et al (2017) Different effects of cardiac and diaphragm function assessed by ultrasound on extubation outcomes in difficult-to-wean patients: a cohort study. BMC Pulmonary Medicine 17:. <https://doi.org/10.1186/s12890-017-0501-8>
12. Ma G, Liao W, Qiu J, et al (2013) N-terminal prohormone B-type natriuretic peptide and weaning outcome in postoperative patients with pulmonary complications. Journal of International Medical Research 41:1612–21
13. Maraghi SE, Hosny M, Samir M, Radwan W (2014) Usage of B-type natriuretic peptide for prediction of weaning outcome by spontaneous breathing trial. Egyptian Journal of Chest Diseases and Tuberculosis 63:671–678. <https://doi.org/10.1016/j.ejcdt.2014.04.003>
14. Martini A, Benedetti B, Menestrina N, et al (2011) Use of NT-proBNP in weaning from mechanical ventilation. Crit Care 15:P162. <https://doi.org/10.1186/cc9582>
15. Mekontso-Dessap A, de Prost N, Girou E, et al (2006) B-type natriuretic peptide and weaning from mechanical ventilation. Intensive Care Medicine 32:1529–1536. <https://doi.org/10.1007/s00134-006-0339-7>
16. Ouanes-Besbes L, Dachraoui F, Ouanes I, et al (2012) NT-proBNP levels at spontaneous breathing trial help in the prediction of post-extubation respiratory distress. Intensive Care Medicine 38:788–795
17. Soummer A, Perbet S, Brisson H, et al (2012) Ultrasound assessment of lung aeration loss during a successful weaning trial predicts postextubation distress*: Critical Care Medicine 40:2064–2072. <https://doi.org/10.1097/CCM.0b013e31824e68ae>
18. Wang Y-T, Fu J-J, Li Y-R, et al (2016) The guiding significance of NT-proBNP and PCT levels in mechanical ventilator of patients with chronic respiratory failure. Eur Rev Med Pharmacol Sci 20:2346–2349
19. Zapata L, Ordonez-Llanos J, Betbesé AJ (2012) Increases in B-type natriuretic peptide for detecting weaning-induced heart failure: reply to Liu et al. Intensive Care Medicine 38:174–174. <https://doi.org/10.1007/s00134-011-2426-7>
20. Zhang Q, Shi ZY, Luo CH, et al (2014) Application of NT-proBNP in ventilator weaning for preterm infants with RDS. Pediatric pulmonology 49:757–63
