# Supplementary material for: Brain natriuretic peptide to predict successful liberation from mechanical ventilation in critically ill patients: a systematic review and meta-analysis
Source: Crit Care. 2020 May 11;24:213. doi: 10.1186/s13054-020-2823-9 (PMC7216735; doi:10.1186/s13054-020-2823-9)

Additional File 4 – Baseline patient characteristics comparison between successful and unsuccessful liberation from mechanical ventilation

Age


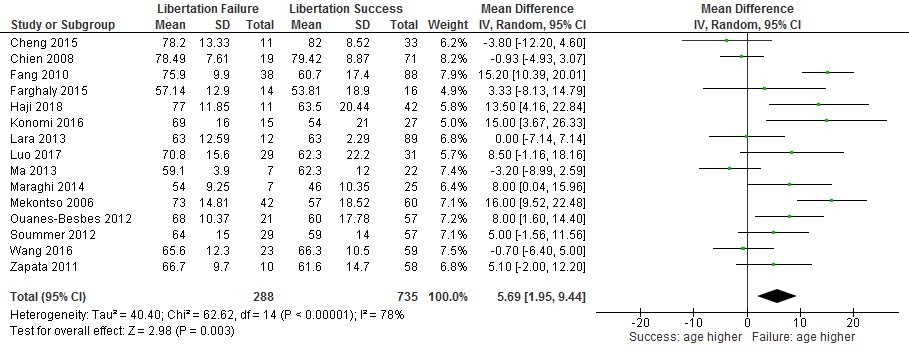


EF


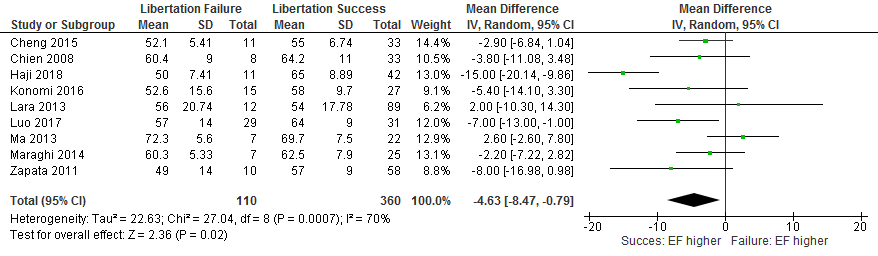


Males


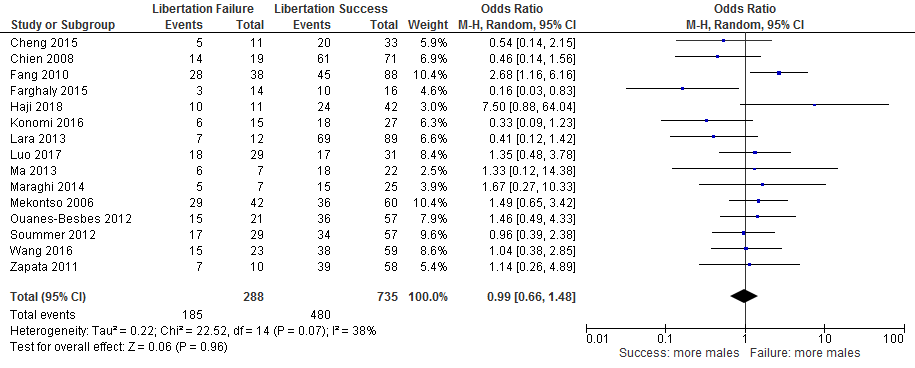


Creat


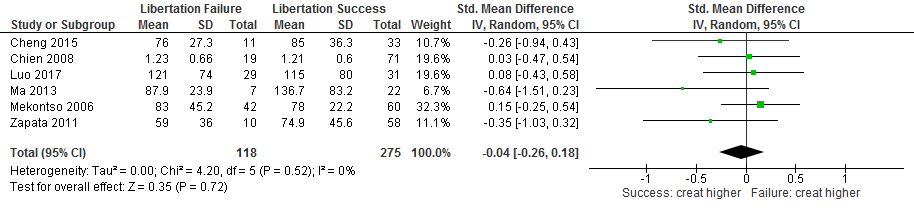


APACHE


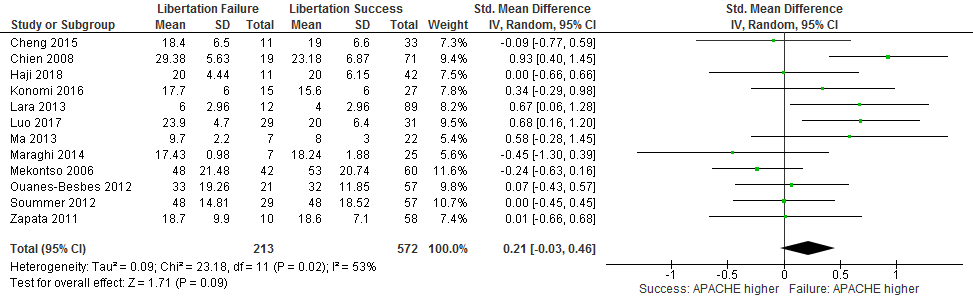


Duration


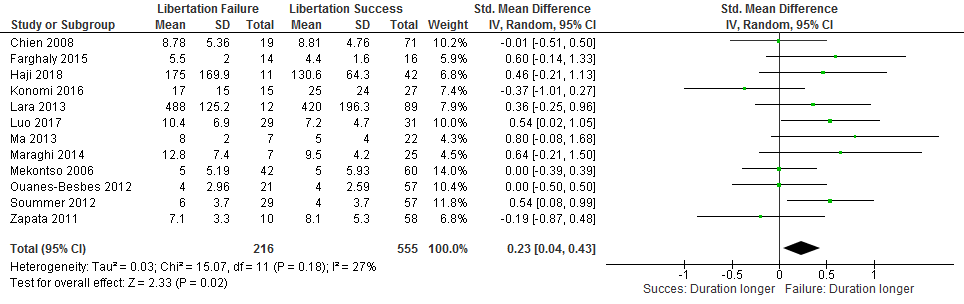


RR24 (b/min)


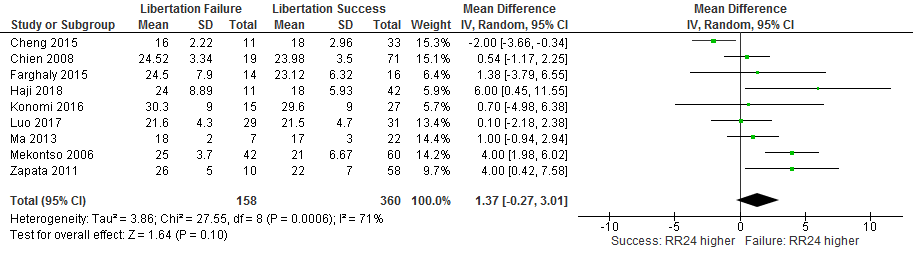


Vt4 (mL)


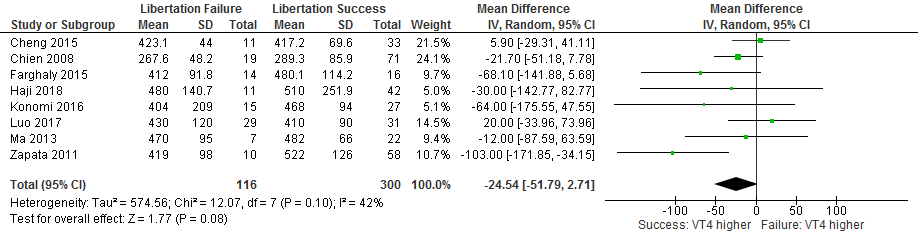


VE (L/min)


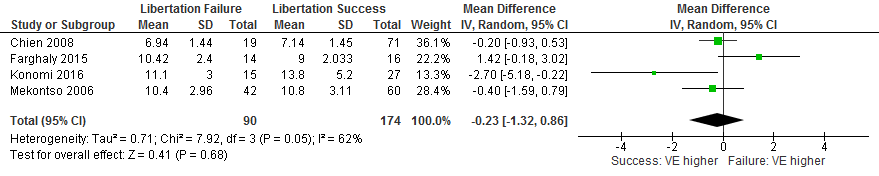


RSBI


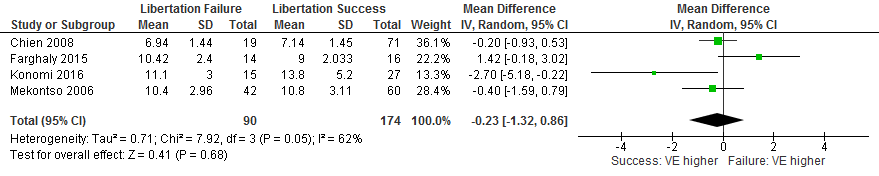


PH


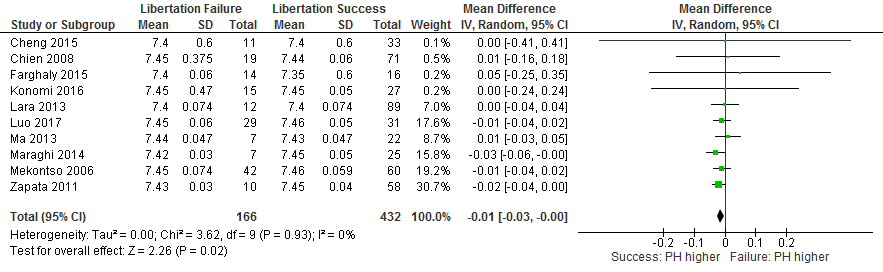


PaO (mmHG)


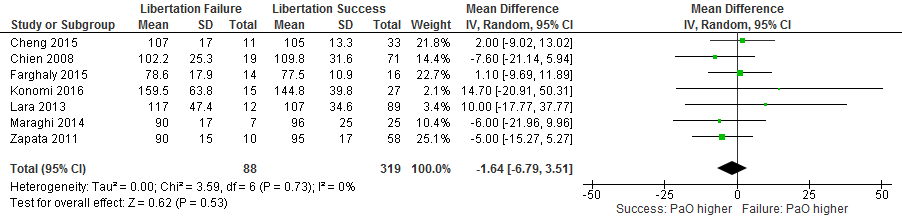


PCO2 (mmHG)


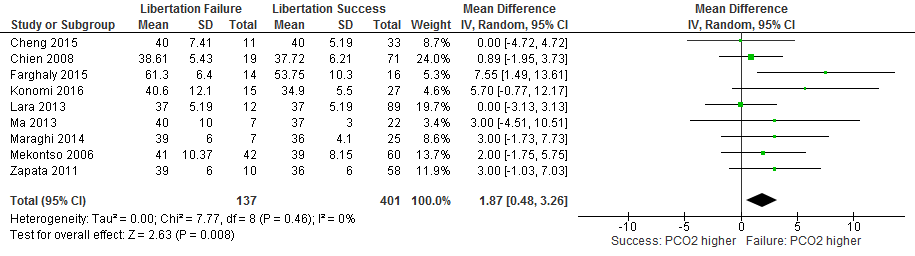


Pa02/fIO227


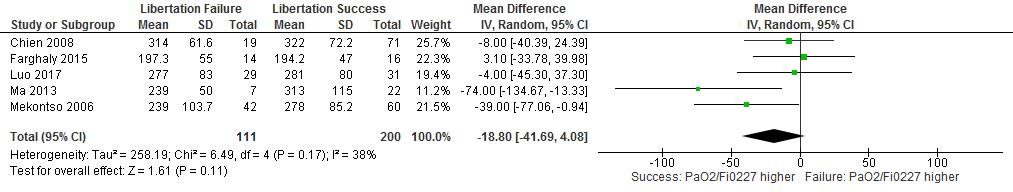

Supplement: Supplementary file 4 — Additional file 4. Baseline patient characteristics comparison between successful and unsuccessful liberation from mechanical ventilation. [file 13054_2020_2823_MOESM4_ESM.docx]
