# Supplementary material for: Brain natriuretic peptide to predict successful liberation from mechanical ventilation in critically ill patients: a systematic review and meta-analysis
Source: Crit Care. 2020 May 11;24:213. doi: 10.1186/s13054-020-2823-9 (PMC7216735; doi:10.1186/s13054-020-2823-9)

Additional File 5 – Moses-Littenberg analysis of either ΔBNP or ΔBNP% methods of measurements in studies that included (group 1) or excluded SBT failure (group 2) from liberation failure analysis.


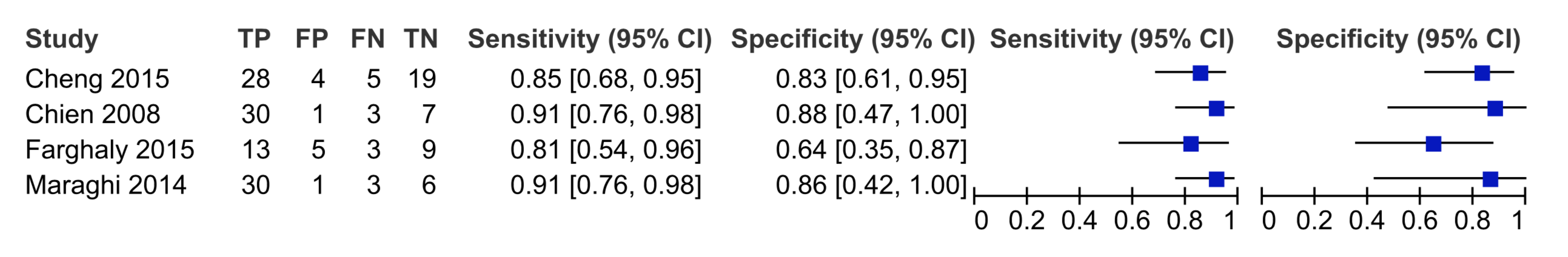


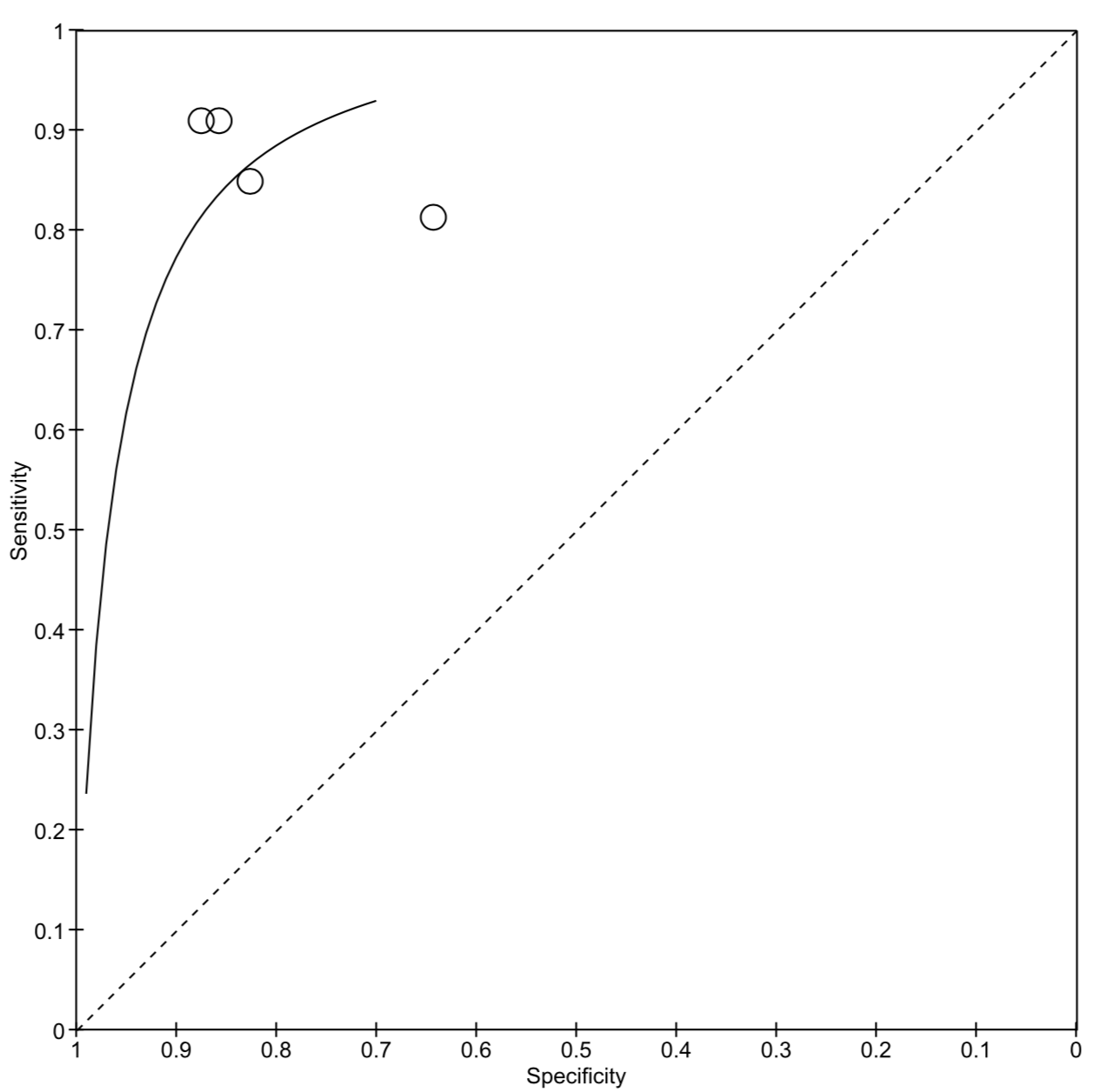

Supplement: Supplementary file 5 — Additional file 5. Moses-Littenberg analysis of either ΔBNP or ΔBNP% methods of measurements in studies that included (group 1) or excluded SBT failure (group 2) from liberation failure analysis. [file 13054_2020_2823_MOESM5_ESM.docx]
